# Human papillomavirus is an important risk factor for esophageal carcinoma in a Chinese population

**DOI:** 10.1007/s00432-022-04322-5

**Published:** 2022-11-17

**Authors:** Wenjun Yuan, Shuying Li, Jun Jia, Li Wang, Yuchao Huang, Minglian Wang, Fei Xie, Jintao Li, Yanzhe Hao

**Affiliations:** 1 Beijing International Science and Technology Cooperation Base of Antivirus Drug, Beijing Key Laboratory of Environmental and Viral Oncology, Faculty of Environment and Life, Beijing University of Technology, 100 Pingleyuan, Chaoyang, Beijing, 100124 People’s Republic of China; 2 Hebei Key Laboratory for Chronic Diseases, North China University of Science and Technology, 21Bohai Dadao, Caofeidian, Tangshan, 063000 Hebei People’s Republic of China; 3 Key Laboratory of Carcinogenesis and Translational Research, Ministry of Education/Beijing, VIP-II Gastrointestinal Cancer Division of Medical Department, Peking University Cancer Hospital & Institute, Beijing, 100142 People’s Republic of China; 4 Department of Materials and Manufacturing, Beijing University of Technology, 100 Pingleyuan, Chaoyang District, Beijing, 100124 People’s Republic of China; 5 Beijing Jinhao Pharmaceutical Co., LTD., Beijing Economic Development Zone, 7 Yuncheng Street, Beijing, People’s Republic of China; 6 NHC Key Laboratory of Medical Virology and Viral Diseases, Chinese Center for Disease Control and Prevention, National Institute for Viral Disease Control and Prevention, Beijing, 100052 China

**Keywords:** Esophageal cancer, Human papillomavirus, Infection, Integration, E6

## Abstract

**Purpose:**

Different types of HPV have been associated with cancer in humans, but the role of HPV in esophageal cancer (EC) is controversial. The purpose of this study was to evaluate the correlation between HPV infection and EC in the Chinese population and to provide the scientific basis for the future prevention, control, early diagnosis, and treatment strategies of EC in China.

**Methods:**

PCR detected HPV infection in 1112 esophageal cancer tissue samples, and 89 HPV-positive samples were detected by genotyping. Proximity ligation assays (PLAs) and immunohistochemistry were used to detect the expression of HPV E6 and E7 proteins. Real-time fluorescent quantitative PCR was used to detect the integration of HPV16 E6. The level of HPV-specific antibody IgG in serum was detected by ELISA and PLA.

**Results:**

The positive rates of HPV L1, HPV16, HPV18, hpv16 + 18 E6 and hpv16/18 E6 in 1,112 EC tissue samples were 77.6%, 41.4%, 27.2%, 14.2% and 55.4% respectively. Multiple HPV subtypes were detected in HPV-positive EC samples. PLA showed that E6 and E7 were expressed in EC109 and formed complexes with p53 and pRb, respectively. Immunohistochemistry showed that the positive rates of hpv16 + 18 E6 and E7 in HPV-positive EC samples were 56.4% and 37.0%, respectively. HPV-DNA integration rate in HPV-positive EC tissues (88.79%) was higher than that in adjacent tissues (54.17%). HPV antibody was found in the serum of EC patients by a serological test.

**Conclusion:**

The study suggests that HPV, especially HPV16 and HPV18, the infection may be a risk factor for EC in the Chinese population and that the E6 protein may play a key role in HPV-associated malignancies. These results may be important for the prevention and treatment of HPV-positive EC in China.

**Supplementary Information:**

The online version contains supplementary material available at 10.1007/s00432-022-04322-5.

## Introduction

Cancers of the esophagus account for over 500,000 cancer deaths annually, representing 5.3% of all global cancer deaths. However, the worldwide regional distribution varies substantially (Bray et al. 2018). Esophageal cancer (EC) usually occurs first in the epithelial layer of the esophagus, and most (about 90%) ECs are squamous cell carcinomas in China. Many EC-related studies have shown variations in EC incidence among countries, and such variation also exists among different areas within the same country. Thus, EC etiology needs to be further determined. Potential risk factors for EC, suggested by previous studies, are excessive intake of alcohol and hot drinks, use of tobacco, nutritional deficiencies, and infectious factors like human papillomavirus (HPV) (Guo et al. 2016; Syrjänen et al. 1982). The exact reasons for the development of EC remain unclear, and different regions may have different EC causes.

Worldwide, the most prominent infectious agents causing cancer are *Helicobacter pylori* (36.3%) and HPV (31.1%) (Szymonowicz and Chen. 2020). HPV is a common, small, double-stranded DNA virus that mainly infects epithelial mucosal cells. More than 200 HPV types have been identified from the genome sequence of L1, including at least 14 high-risk types that may lead to cancer (Doorbar et al. 2012). The key to carcinogenesis caused by high-risk HPVs is the integration of HPV DNA into the host genome, which leads to the upregulation of viral oncoproteins, especially E6 and E7, causing excessive proliferation of host cells. Various studies have confirmed the role of high-risk HPV in the etiology of cervical cancer (Muñoz et al. 1994; Walboomers et al. 1999).

In recent years, HPV has been considered a risk factor, as well as an indicator for better survival, of patients with head and neck squamous cell carcinoma (Wagner et al. 2017). The relationship between HPV infection and squamous cell tumors dates back to the early 1980s when Syrjänen first proposed the hypothesis that HPV is related to EC carcinogenesis. Later studies reporting on the relationship between HPV infection and EC occurrence found that high-risk HPV16 and HPV18 infections are possible risk factors for the occurrence of EC (Pantham et al. 2017; Wang et al. 2016a, 2016b; Xi et al. 2016; Yahyapour et al. 2016; Zang et al. 2015), but the results of various studies are quite different. A study from Anyang (a region in China) has confirmed the presence of the HPV16 genome as an important infectious factor for high EC incidence (Li et al. 2001). Likewise, Shantou is a high-risk area for EC in China, and the infection and integration rates of HPV DNA in patients with EC are high, suggesting that HPV infection is a potential risk factor for EC (Zhang et al. 2011b). Geographic location likely accounts for the majority of variations in HPV prevalence especially in high-risk areas (Ludmir et al. 2015). However, other studies have shown that HPV is not associated with EC (Antunes et al. 2013; Gao et al. 2006; Kanaan et al. 2020). A Brazilian study examining 87 samples of patients with EC did not find an association with HPV (Bognár et al. 2018). Another study from Linxian using balloon cytology examination did not consider HPV a major risk factor but suggested further exploration of the role of HPVs in ESCC (Sultana et al. 2020). A meta-analysis from China regarding HPV18 prevalence indicated moderate HPV prevalence in EC and mentioned the need for further studies to clarify the role of HPVs in EC etiology (Guo et al. 2016). Another study suggested the role of HPV in causing EC only in high-risk areas like Asian countries (Ludmir et al. 2015). This may be due to differences in geographic regions, ethnic groups, tissue samples, and testing methods used.

It has been suggested that HPV-positive tumors represent an independent entity different from HPV-negative tumors, as they differ in manifestations and treatment responses. This has a direct impact on tumor treatment and prognosis (O'Sullivan et al. 2013; Rittà et al. 2009). To obtain more reliable data, tissue samples from 1112 EC patients from different regions of China were collected in this study. Different methods were used to process the same batch of samples under the same conditions to avoid the error of results caused by geographical regions, ethnic groups, small sample numbers and differences in detection methods used. The significance of HPV in the development of EC was analyzed in detail, which is of great significance for the prevention and treatment of HPV-positive EC.

## Methods

### Detection of HPV DNA in tissues of esophageal carcinoma

Esophageal squamous cell carcinoma (ESCC) tissue specimens were taken from 10 high-risk area pathology departments in China from 2009 to 2012 (Linzhou, Huaihe River Basin, Anyang, Cixian, Jieyang, Yanting, Luoyang, Puyang, Tangshan, Baoding). 1,112 histologically confirmed ESCC specimens (FFPE tissue blocks) were sectioned and DNA was extracted. The specific process refers to our previous research (Mehryar et al. 2015). DNA extraction was amplified using the PCR kit (Takara, Japan). The primer information of HPV DNA amplification is shown in Table 1. Water and HPV-positive cervical cell DNA serve as negative and positive controls, respectively. The specific process of PCR was referred to Jacobs (Jacobs et al. 1997). PCR products were detected by 2% agarose gel electrophoresis containing 0.01% Gold View and analyzed by gel imager after electrophoresis.

**Table 1:** Primers used in PCR amplification of human papillomavirus DNA

Primer	Sequence (5’–3’)	Annealing temperature (℃)	PCR product (bp)
PC04 ^a^	CAACTTCATCCACGTTCACC	62	150
GH20	GAAGAGCCAAGGACAGGTAC		
GP5 + ^b^	TTTGTTACTGTGGTAGATACTAC	55	150
GP6 +	CTTATACTAAATGTCAAATAAAAAG		
HPV16-E6(F)	GCAAGCAACAGTTACTGCGA	60	321
HPV16-E6(R)	CAACAAGACATACATCGACC		
HPV18-E6(F)	CACTTCACTGCAAGACATAGA	55	322
HPV18-E6(R)	GTTGTGAAATCGTCGTTTTTCA		

^a^PC04/GH20 is a β -globin specific primer used to detect the quality of extracted DNA

^b^GP5 + /GP6 + is a universal primer for detecting HPV L1

To identify HPV types and any sequence variations, PCR products from all samples were sequenced and analyzed using the T7 Sequence Version 2.0 DNA PCR product sequencing Kit (Affymetrix, USA). The nucleotide sequence was then determined using BLAST (https://blast.ncbi.nlm.nih.gov/Blast.cgi).

### Detection of HPV subtypes in esophageal carcinoma samples

Eighty nine samples were randomly used for further genotyping of HPV. 21 HPV GenoArray Diagnostic Kit (Guangzhou Hybribio Biotech Ltd, China) was used for rapid and accurate HPV genotyping. It can identify 21 HPV including 15 high-risk types (HPV 16, 18, 31, 33, 35, 39, 45, 51, 52, 53, 56, 58, 59, 66, 68) and 6 low-risk types (HPV 6, 11, 42, 43, 44, 81).

### In situ PLA for detection of HPV cancer protein in esophageal carcinoma cells

Cell culture and preparation of centrifuge cell smear. In the experimental group, EC109 cell lines infected with HPV18 were suspended in PBS at medium density, then coated on glass slides, dried at room temperature and fixed with poly-oxy-methylene (4%). The control group was treated with normal HEK 293 cells in the same way.

In situ PLA (Proximity ligation assay). In the experiment, each protein molecule was detected by a monoclonal antibody for p53, pRb, HPV18 E6 and E7. HPV18 E6-p53 protein complex was detected by Rabbit anti HPV18 E6 antibody and mouse anti p53 antibody, and HPV18 E7-pRb protein complex was detected by Rabbit anti HPV18 E7 antibody and mouse anti p53 antibody. The PLA test kit was purchased from Olink Bioscience (Uppsala, Sweden) and the specific operation was carried out by the manufacturer's operating procedures. Then the results were observed by laser confocal microscope.

### Immunohistochemistry study (IHC)

HPV16+18 E6 and HPV16+18 E7 monoclonal antibodies were used to detect cancer tissues. The antibodies used to detect HPV-18 and 16 are mouse monoclonal antibodies. We used the same procedure previously used in the laboratory (Mehryar et al. 2015). The immunohistochemical interpretation was performed independently by two laboratory staff under double-blind conditions. To understand the positive staining intensity and location of E6 and E7 proteins in cells, five visual fields were selected under a 40x microscope. If there are three or more areas with nuclei or cytoplasm, it is judged that brown granules or brown staining are a positive result.

### Detection of HPV DNA Integration in esophageal carcinoma cells

One hundred and sixteen cancer tissues and 72 adjacent tissues were randomly selected for real-time fluorescent quantitative PCR to detect the integration of HPV DNA in esophageal cancer. The research is based on the following assumptions: the special region of the E2 open reading frame (ORF), which is most often missing during HPV integration, such as HPV exists in the form of free type, and the proportion of E2 and E6 copies is equal. If HPV is integrated into the host chromosome, E2 is deleted, and the copy number ratio of E2 to E6 is zero; HPV exists in a mixed form of free and integrated, and the copy number of E2 is less than E6. Refer to our previous article (Li et al. 2017) for specific methods. According to the HPV16 gene sequence (K02718), different regional loci of E2 and E6 genes were selected to design HPV16-E2 and E6 primers (Table 2). HPV16-E2 and E6 were detected by the same amount of DNA in each specimen, and the ratio of E2 to E6 was calculated to determine the integration status of HPV. PCR amplification was performed using EvaGreen^®^ Dye (Biotium, USA) in the RotorGene fluorescent PCR system.

**Table 2:** Sequences of real-time qPCR primers

Primer	Sequences (5'–3')	Position	Amplicon size(bp)
HPV16 E2 Primers ^a^			
HPV16 E2-1	ATGGAGACTCTTTGCCAACG	2755..2774	188
HPV16 E2-2	GCCAGTGTTGGCACCACTTGGTGG	2919..2942	
HPV16 E2-3	AGCAGCAACGAAGTATCCTCTC	3355..3376	172
HPV16 E2-4	GCAACAACTTAGTGGTGTGGC	3506..3526	
HPV16 E2-5	GTAACACTACACCCATAGTAC	3602..3622	185
HPV16 E2-6	GTCACGTTGCCATTCACTATC	3766..3786	
HPV16 E6 Primers			
HPV16 E6-5	CTGCGACGTGAGGTATATGAC	214..235	320
HPV16 E6-6	TTGATGATCTGCAACAAGACATAC	510..533	
β-actin Primers			
Forward	TCACCCACACTGTGCCCATCT	459..560	290
Reverse	GAACCGCTCATTGCCAATGG	819..838	

^a^HPV16 E2 is the primer of three E2 gene regions of HPV16

### Antibody detection of HPV in serum samples of esophageal carcinoma

The sera of 76 patients with esophageal cancer and 149 physical examiners were randomly selected. Combined with a series of serological diagnostic methods for nasopharyngeal carcinoma established by academician Zeng Yi of our laboratory in the 1970s (Zeng et al. 1982, 1983b), HPV-related IgG antibody was detected in serum combined with PLA. Enzyme-linked immunosorbent assay (ELISA) is also used to detect HPV antibody levels in serum.

Serum HPV antibody was detected by PLA. Duolink II Fluorescence reagents (Olink Bioscience, Sweden) were used to detect HPV-related antibodies in serum. HPV18-infected EC109 cells were detected by nasopharyngeal carcinoma detection method and PLA technology. Normal HEK293 cells are used as the negative control. Please refer to the equipment manual for detailed operation.

HPV antibody was detected by ELISA. The human papillomavirus IgG antibody detection kit (Beijing Founder Biotechnology Co. LTD, China) was used to detect human papillomavirus-related IgG antibody levels in serum. Please refer to the instructions for specific operations.

### Statistical analysis

SPSS23.0 statistical software was used for statistical analysis of the results, and the correlation between HPV-related proteins and esophageal cancer was analyzed. The *P* value of the tests had to be < *0.05* to be statistically significant. The correlation coefficient of the lollipop chart and multi-type pie chart by the free online data analysis and visualization platform https://www.bioinformatics.com.cn.

## Results

### Detection of HPV DNA in esophageal carcinoma tissue

**Fig. 1:**
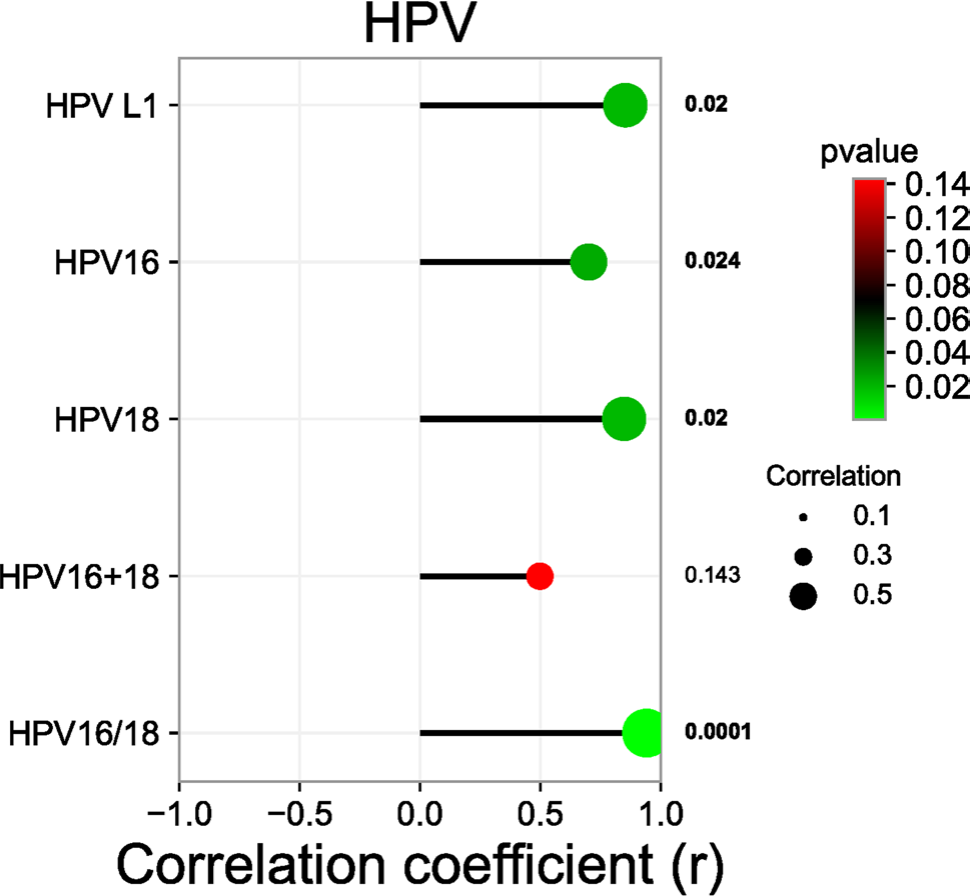
Correlation analysis between human papillomavirus (HPV) and esophageal cancer

Table 3 summarizes the PCR results of all 1,112 ESCC samples included in this study. PCR amplification of extracted DNA with β-actin as an internal reference showed that more than 98% of ESCC samples had good DNA quality and all displayed differentiated 150-bp DNA fragments (Online Resource 1, Fig. S1a). PCR amplification was performed with 1,027 paraffin-embedded specimens of esophageal from different regions in China (Linzhou, Huaihe River Basin, Anyang, Cixian, Jieyang, Yanting, Luoyang, Puyang, Tangshan, Baoding) using universal HPV L1 primers (Online Resource 1, Fig. S1b). Of these samples, 797 (77.6%) L1-positive samples were identified by agarose gel assay. Finally, HPV16- and HPV18-specific primers were used for PCR amplification (Table 3). Both PCRs produced high-intensity bands of the HPV types 16 and 18 with expected sizes (350 bp; Online Resource 1, Fig. S1c and d). The positive rates of HPV16, HPV18, HPV16+18 E6, and HPV16/18 E6 were 460/1,112 (41.4%), 302/1,112 (27.2%), 146/1,027 (14.2%), and 616/1,112 (55.4%), respectively (Table 3). Correlation analysis of the 1,112 ESCC samples showed that both HPV16 and HPV18 were correlated with EC incidence (Pearson’s correlation coefficients: *R*=0.701, *P*=0.024 and *R*=0.848, *P*=0.26, respectively). HPV16/18 showed the strongest correlation with EC (*R*=0.941, *P*=0.0001; Fig. 1).

**Table 3:** PCR detection frequency of different human papillomavirus (HPV) types in esophageal cancer samples from ten high-risk areas of China

HPV type	Total no. of cases tested	HPV-positive cases (no.)	HPV-positive cases (%)
HPV L1	1027	797	77.6
HPV16	1112	460	41.4
HPV18	1112	302	27.2
HPV16 + 18 E6	1027	146	14.2
HPV16/18 E6	1112	616	55.4

**Fig. 2:**
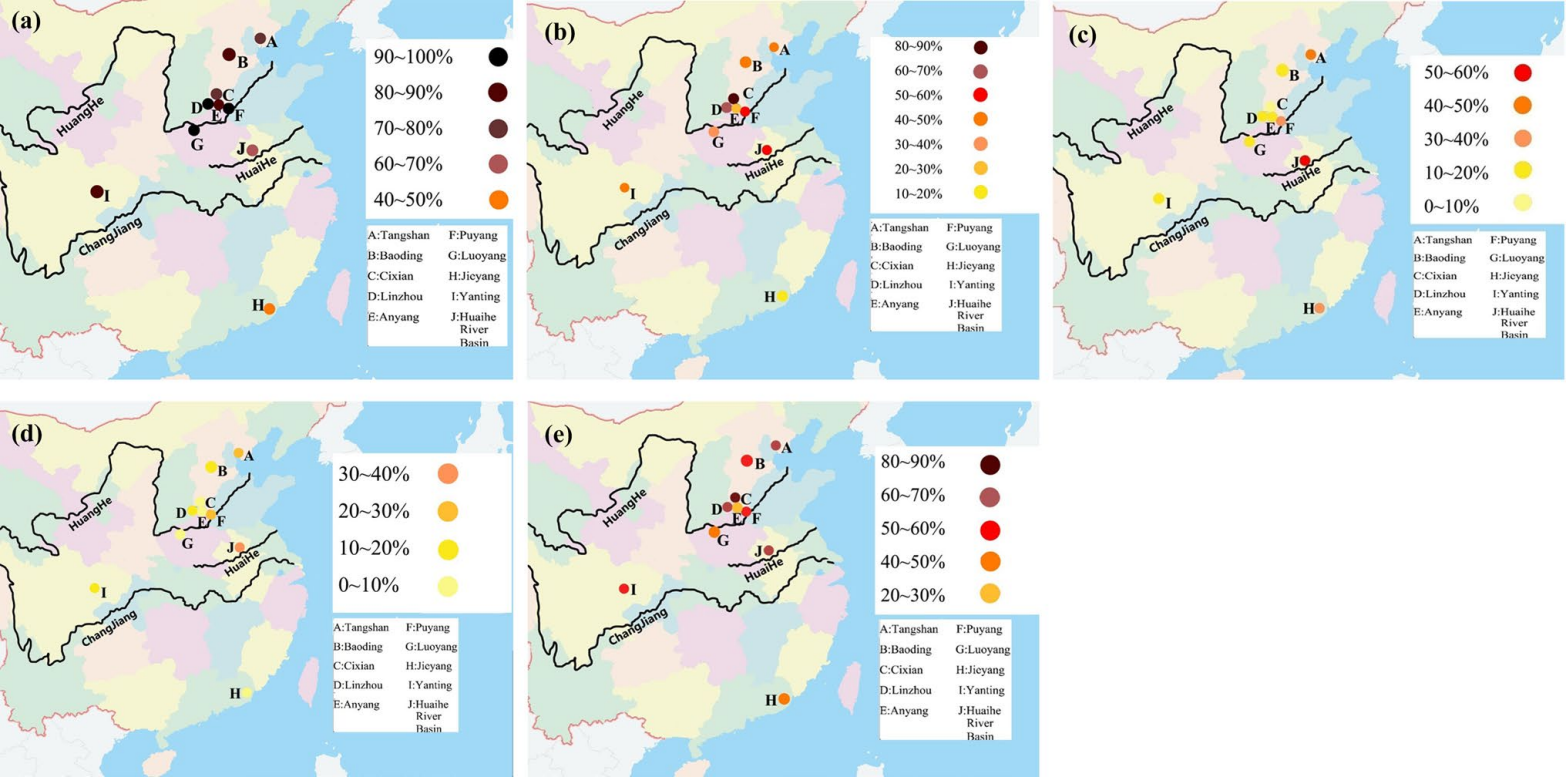
Prevalence rates (in %) of human papillomavirus (HPV) types detected by PCR in different regions of China. **a** HPV L1, **b** HPV16, **c** HPV18, **d** HPV16 + 18, and **e** HPV16/18

**Fig. 3:**
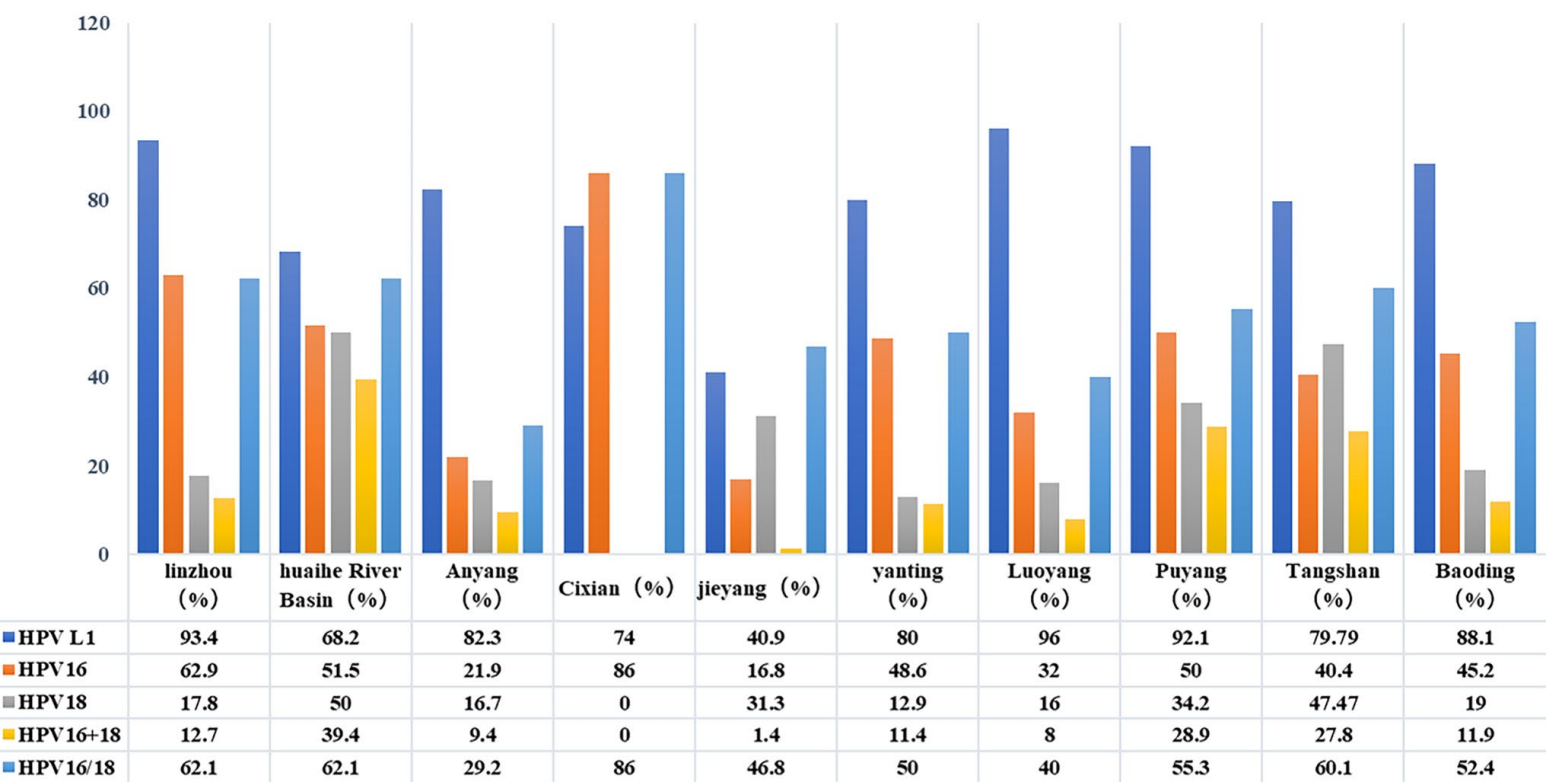
Prevalence rates of human papillomavirus (HPV) types detected by PCR in esophageal cancer samples from ten high-risk areas of China

We also analyzed HPV L1 DNA results from the 1,112 samples regarding differences among the examined ten regions. Luoyang had the highest HPV L1 positive rate (96.0%), followed by Linzhou (93.4%) and (Puyang 92.1%), and Jieyang had the lowest rate (40.9%). The positive rate of HPV16 was higher in Cixian, Linzhou, and Huaihe River Basin, with positive rates ranging from 16% to 86%. The positive rates of HPV18 in Huaihe River Basin, Tangshan, and Puyang were 50.0%, 47.47%, and 34.2% respectively. The positive rate of HPV16 E6 and HPV18 E6 was 39.4% in Huaihe River Basin, 28.9% in Puyang, and 27.8% in Tangshan. The positive rate of HPV16/18 was the highest in Cixian (86.0%), followed by Linzhou (68.0%), and Huaihe River Basin (62.1%). The HPV prevalence rates in ten examined high-risk areas of China and the details of patients with EC positive for different HPV types are shown in Figs. 2and 3. In EC tissues, the detection rate appears to be higher for HPV16 than for HPV18.

### Detection of HPV subtypes in esophageal carcinoma samples

**Fig. 4:**
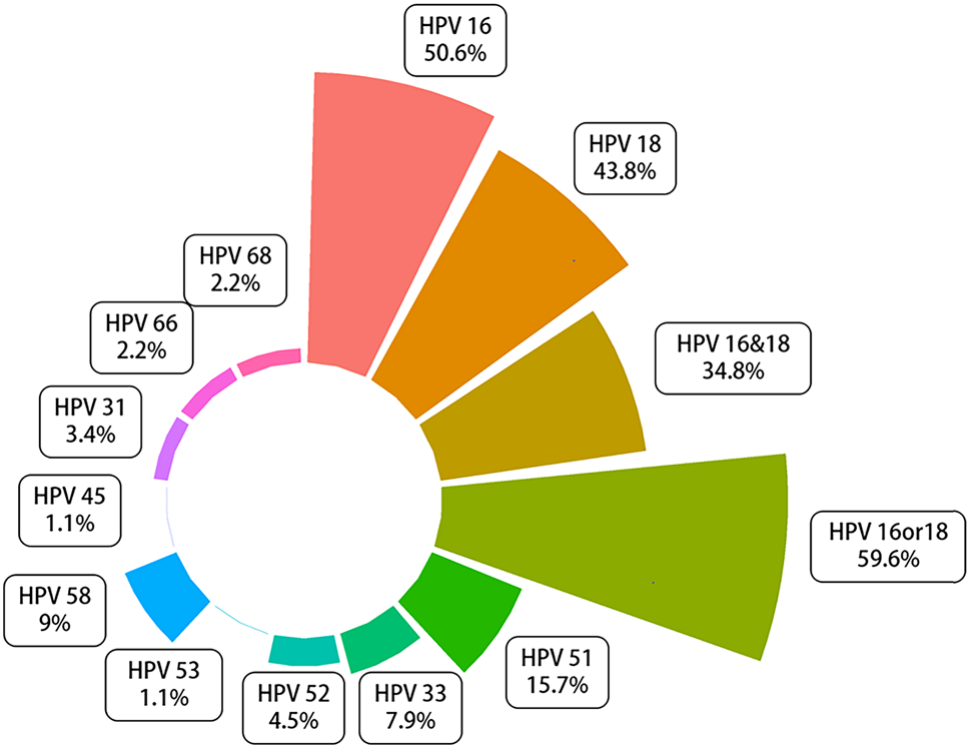
Presence of human papillomavirus (HPV) subtypes in esophageal cancer samples

We randomly selected 89 specimens of HPV-positive EC for HPV DNA, and their HPV virus types were detected using a kit. The positive rates of HPV16 and HPV18 were in this subpopulation 50.6% and 43.8%, respectively. HPV16 and HPV18 were simultaneously present in some samples (34.8%), and the positive detection rate of HPV16 and HPV18 was high. HPV51 was also widely distributed in EC tissues (positive rate: 15.7%). HPV45, 33, 52, 53, 58, 45, 31, 66, 68, and other types were also found (Fig. 4), suggesting that HPV may play an important role in the occurrence and development of EC.

### Detection of HPV cancer protein in esophageal carcinoma cells

**Fig. 5:**
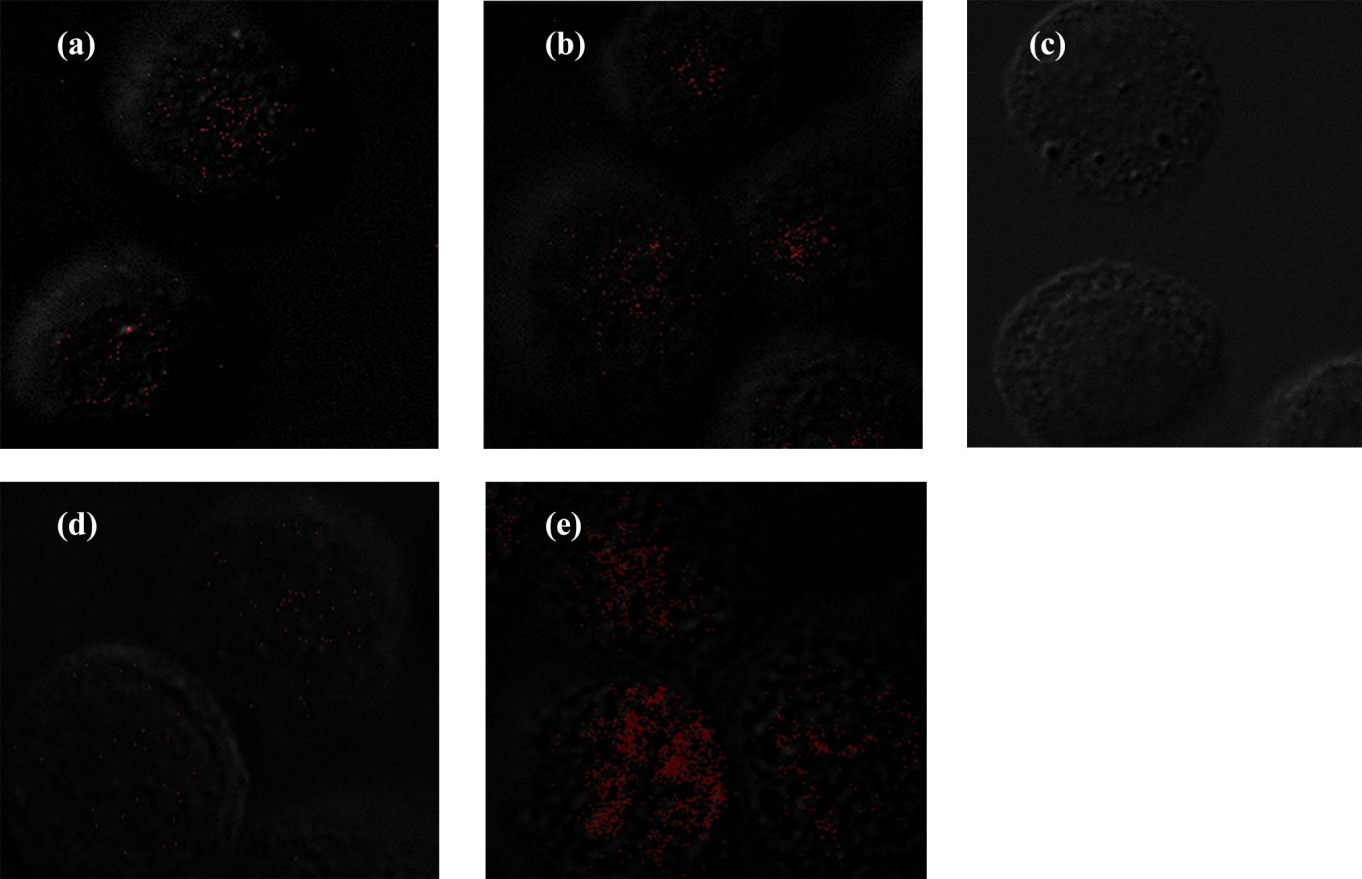
**a** Detection of interactions between pRb and HPV18 E7 protein in EC109 cells. **b** Detection of interactions between p53 and HPV18 E6 protein in EC109 cells. **c** Negative control. **d** Detection of HPV18 E7 protein alone in EC109 cells. **e** Detection of HPV18 E6 protein alone in EC109 cells

In situ PLAs were used to detect the expression of HPV18 onco-proteins E6, E7, E6–p53, and E7–pRb complex in the tumor cell line EC109 infected with HPV18 (Fig. 5). Target protein complexes were detected as red fluorescent spots. The fluorescence signal was stronger in EC109 cells infected with HPV18 E6 than in cells infected with HPV18 E7 (Fig. 5d and e). The results confirmed that HPV protein is stably expressed in infected EC host cells, and the host target protein interacting with HPV was identified.

### Immunohistochemistry analysis

**Fig. 6:**
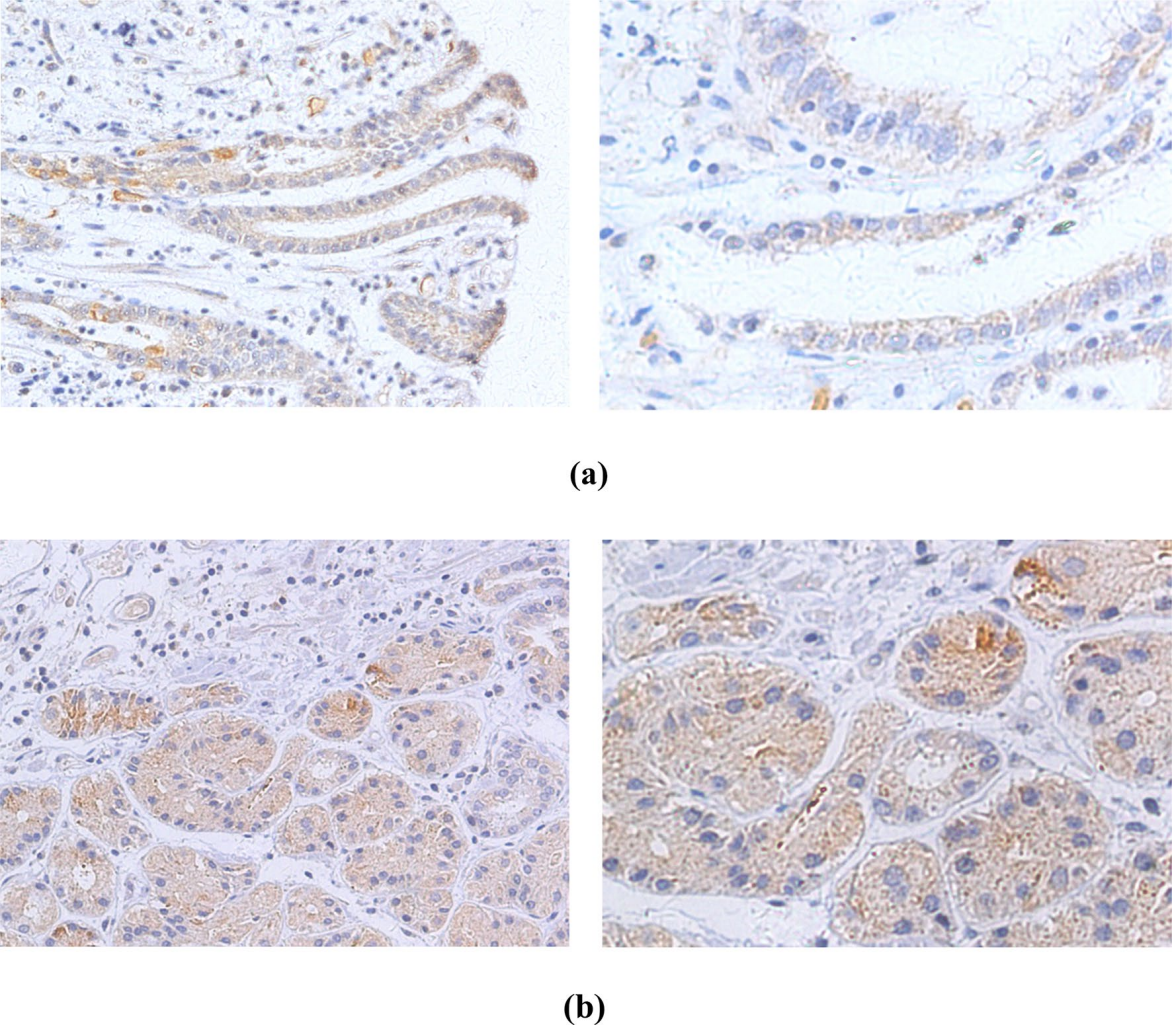

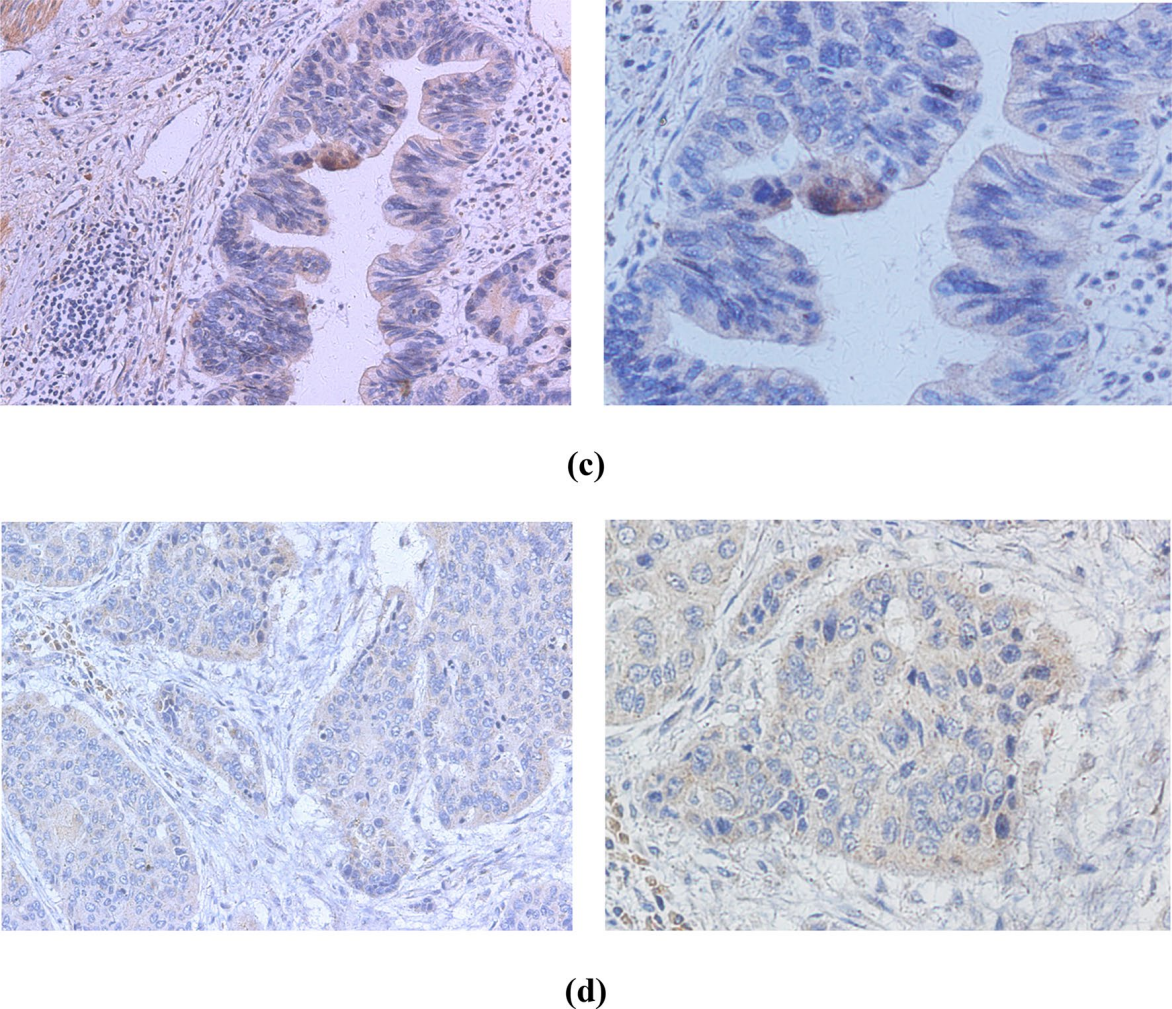
Immunohistochemical stainings are positive for different human papillomavirus types. **a** HPV16 E6. **b** HPV18 E6. **c** HPV16 E7. **d** HPV18 E7. (left, 200 × ; right, 400 ×)

Immunohistochemistry (IHC) was performed in 275 EC cases positive for HPV DNA. HPV-specific proteins, including monoclonal antibodies against HPV16+18 E6 and HPV16+18 E7, were used to detect cancer tissues. Representative IHC images are shown in Fig. 6. The immunohistochemical results showed that the expression levels of HPV16+18 E6 were higher than those of HPV16+18 E7 (56.4% and 37.0%, respectively; Table 4).

**Table 4:** Summary of human papillomavirus HPV16 + 18 E6 and E7 immunohistochemical stainings

HPV type	Samples (no.)	Positive no. (%)	Negative. (%)
HPV16 + 18 E6	140	79 (56.4)	61 (43.6)
HPV16 + 18 E7	135	50 (37.0)	85 (63.0)

### Detection of HPV DNA integration in esophageal carcinoma cells

Of 116 HPV16 E6-positive EC specimens, 103 (88.8%) were positive for E2 gene integration, whereas, among 72 adjacent tissue specimens, 39 (54.2%) were positive for E2 gene integration (Table 5). The integration rate of the HPV genome into host genes is very high in EC, even in adjacent tissues.

**Table 5:** HPV16 gene integration in HPV16-positive esophageal cancer samples and para-cancerous tissue

	Positive for HPV16 E6	Integrated ratio (%)	Significance ^a^
Cancerous tissue	103	(103/116) 88.79	*P* < *0.05*
Paracancerous tissue	39	(39/72) 54.17	

^a^ *P* < *0.05*, the difference between groups was significant and statistically significant

### Antibody detection of HPV in serum samples of patients with esophageal carcinoma

**Fig. 7:**
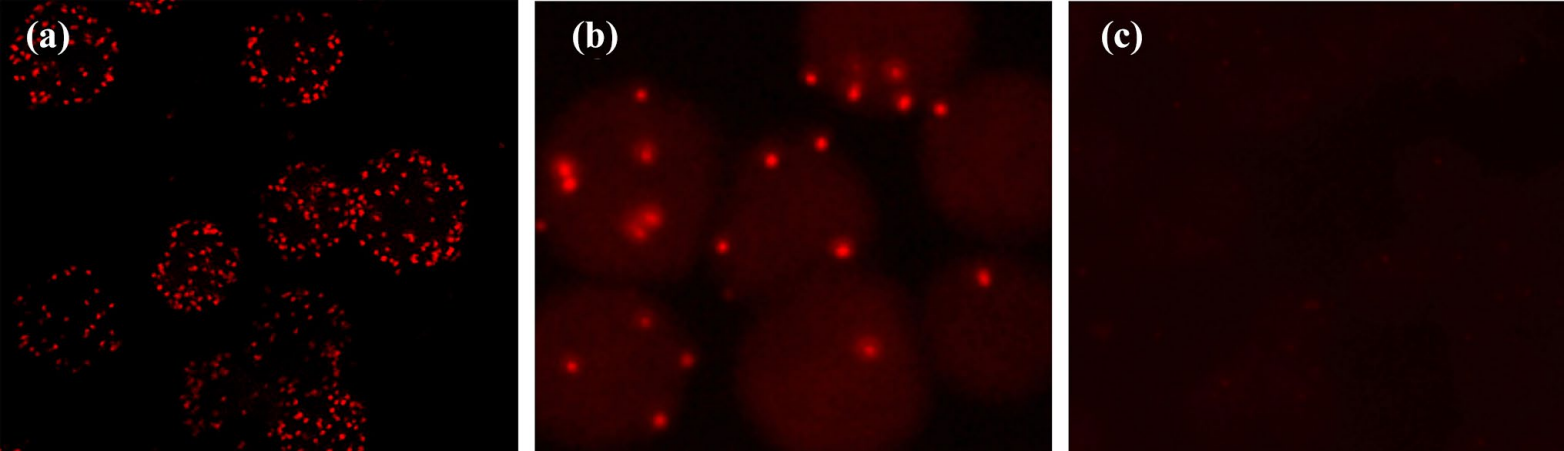
**a** Seropositive reaction in a patient with esophageal cancer. **b** Seropositive reaction in a participant from the normal examination group. **c** Negative control group

We used PLAs to detect HPV-related IgG antibody levels in the serum of EC patients. The results showed that all EC patients and healthy controls were positive, but the number of red fluorescent spots in the serum was different (Fig. 7a and b), and the serum of the negative control group did not show a positive reaction (Fig. 7c). We used the PLAs method established by ourselves. The analysis results showed that although the sensitivity was high, it did not show significant differences between tumor patients and healthy people. Therefore, we chose the routine serological antibody detection method ELISA to detect HPV IgG levels in the same batch of serum samples. A total of 37 EC patients (48.7%) were IgG positive, while 79 healthy controls (53.0%) were IgG negative, but this difference was not statistically significant (Table 6).

**Table 6:** ELISA results of HPV-related IgG antibodies in serum

	Positive rate (%)	Significance ^a^
Patients with esophageal cancer	37/76 (48.7%)	*P* > 0.05
Healthy control group	79/194 (53.0%)	

^a^ *P* > 0.05, there was no statistical significance

## Discussion

EC is the most aggressive malignant tumor worldwide. Like in other cancer types, EC development is a complex process involving multiple steps and etiological factors. In some countries with low ESCC prevalence, the main causative factors are the use of tobacco and alcohol. However, in ESCC high-risk areas like China and Iran, only a small number of EC cases are found to be associated with the use of tobacco or alcohol (Islami et al. 2004; Pourshams et al. 2005; Tran et al. 2005). Therefore, other risk factors should be investigated to identify the reasons responsible for the high EC incidence in these regions. Factors involved in EC pathology could be the intake of hot drinks, contaminated water and food, deficiency of essential nutrients, and infectious agents like HPV (Ghaffar et al. 2018; Guo et al. 2016; Zhang et al. 2011b). High-risk HPV types have been observed in at least 90% of cervical cancers and approximately 20% of head and neck cancers (Liu et al. 2013). Many studies from different parts of the world have shown the presence of the HPV genome in DNA isolated from EC tissues; however, the results vary (Liu et al. 2013, 2017; Wang et al. 2016a; Yahyapour et al. 2016; Yong et al. 2013; Zhang et al. 2015). The strongest evidence for an association between ESCC and HPV was found in a meta-analysis demonstrating that HPV infection leads to a three-fold increase in ESCC risk (Liyanage et al. 2013). Although most studies have shown the presence of high-risk HPV types in a variable proportion of cases, HPV has not been confirmed as an etiologic agent of EC, even in highly prevalent regions.

In this study, tissue samples were collected from 1112 pathologically diagnosed ESCC patients in ten areas with high EC incidence in China. The HPV detection rate was 77.6% (Table 1 and Fig. 3), and this result is consistent with findings of previous studies in Tangshan and Shantou (79% and 77.4%, respectively) (Mehryar et al. 2015; Zhang et al. 2011b). As shown in

areas with high EC incidence in China are mainly concentrated in the Taihang Mountains. The positive rate of HPV16 in EC tissue samples is twice that of HPV18, which is quite different from findings in cervical cancer samples. To analyze the types of HPV infections, we subsampled HPV-positive EC tissues. The positive rates of HPV16 and HPV18 were the highest, with HPV51, HPV33, and HPV58 also accounting for a high proportion (Fig. 4). Even multiple HPV types were simultaneously detected in some samples. A recent meta-analysis found that the summary OR of ESCC was 4.04 (95% CI, 2.81–5.82%) for Asian patients with HPV16/18 infection compared with those with no HPV infection (Petrelli et al. 2021). This is consistent with the results of our previous study (HPV as a risk factor for esophageal squamous cell carcinoma, OR 4.20, 95% CI, 3.08–5.74, *P* < 0.00001) (Liu et al. 2013). Our correlation analysis showed that HPV16/18 was strongly correlated with EC (Fig. 1). This suggests that HPV is prevalent in patients with EC, particularly HPV16 and HPV18, of which HPV16 seems to be more easily detected in EC tissue (Table 3). HPVs, especially HPV16, may play an important role in the development of EC.

E7 and E6 onco-proteins have been found to specifically interact with specific tumor suppressor proteins, such as pRb and p53 (Javadi et al. 2018), to inhibit the tumor suppressor function of p53 and pRb. We used the Duolink^®^ PLA technique to observe the expression of p53, pRb, HPV18 E6 and E7 in HPV18-infected EC109 cells. This technique recognizes the target protein through a pair of monoclonal (polyclonal) antibodies labeled with single-stranded DNA. Only when a pair of probes are close enough (<40 nm), a pair of probes labeled with antibodies will combine to form a ring, which is reflected by an increase in fluorescence intensity. We observed that E7–pRb and E6–p53 protein complexes were highly prevalent in EC109 cells. This not only directly proves the interaction between the two proteins, but also indicates that the cells expressing HPV E6 and E7 proteins are carcinogenic. Therefore, we believe that HPV-positive cells or tissues play an important role in carcinogenesis. We also found that the expression of E6 was stronger than E7 after HPV18 infected EC109 cells (Fig. 5). Similarly, IHC studies on the expression of HPV16+18 E6/E7 proteins showed that in EC samples, the positive rate of HPV16+18 E6 was also higher than that of HPV16+18 E7, which is consistent with the PLA results of the current study. HPV E6 protein appears to be more likely than E7 to enter EC cells and plays a biological role, but this has not been previously reported. The specific reasons for this observation are not clear, and further research is needed to explain this phenomenon.

It is well known that the integration of human papillomavirus DNA into the human genome is a key driver of cervical cancer (Cao et al. 2020). Therefore, to study whether HPV DNA integration is an important factor affecting the occurrence of EC, we detected the integration of HPV16 E6 in EC cells and found that the integration rate of HPV DNA in EC tissues (88.8%) was higher than that in adjacent tissues (54.2%). This high integration rate is similar to the results of a previous study in the Shantou region (integration rate, 93.4%) (Zhang et al. 2011b). Another study from China also showed similar results, suggesting that the integration of the virus into the host genome may be a key event to promote and initiate the occurrence of EC (Si et al. 2005). HPV integration leads to overexpression of HPV oncogenes (E6/E7), which affects key cell pathways, such as cell cycle and apoptosis, leading to cell carcinogenesis.

One of our previous studies investigated infection and integration of high-risk types of HPV in different cancer cell lines. HPV DNA is inserted into EC9706, EC109 and other cell lines, but only part of the integrated DNA is transcribed into mRNA (Liu et al. 2015). The integration sites of HPV DNA into host chromosomes are not random. For example, HPV16 DNA tends to integrate into 13q21 (cervical cancer cell lines), whereas HPV18 DNA tends to integrate into 8q24 (EC109 cell line), which is a genetic desert area and changes in these areas often lead to tumors (Diao et al. 2015; Zhang et al. 2011a). With the pathological stage of EC development, the prevalence of HPV16 integration is very high. At the pathological stage of EC development, the prevalence of HPV16 integration is very high. The results suggest that the integration of HPV16+18 DNA into the host genome plays a key role in the malignant transformation and carcinogenesis of EC cells (Li et al. 2018, 2017). This motivates me to study the relationship between HPV and EC.

Serological detection is another important method for early tumor diagnosis. We further investigated the relationship between HPV and EC using serological methods like Duolink^®^ PLA and ELISA. The Duolink^®^ PLA results showed that a high proportion of HPV-related IgGs and IgAs existed in the serum of patients with EC, indicating that most patients with EC had been infected with HPV. However, red spots were also detected in the serum of normal people, indicating that the sensitivity of this method is very high and that HPV infection and the presence of corresponding antibodies is a common phenomenon. The fluorescent spot density produced by each person’s serum is also different, which may be related to differences in personal constitution. HPV-specific ELISAs showed no significant differences between HPV-positive patients with EC and people with normal physical examination, and the antibody level was higher than those previously reported. Similarly, serological tests for HPV in patients with cervical cancer are not ideal (Singini et al. 2022). These findings suggest that HPV infection is widespread in the general population and induces immune responses, but cannot be used as a diagnostic indicator.

The results of this study are consistent with those of previous studies regarding the correlation between HPV infection and EC. Some controversial studies show no association between HPV and EC (Antunes et al. 2013; Kanaan et al. 2020; Pastrez et al. 2017; Zeng et al. 1983a). Several reasons may explain this discrepancy. EC is a complex multifactorial disease. Several factors distinguishing different countries or different parts of the same country, including the regional environment, eating habits, or specific customs, may contribute to diverging results. Furthermore, the number of samples used in each study is limited, and the sensitivity of sample preparation and evaluation methods, including the quality of DNA and tissue samples used may vary greatly. Most importantly, HPV viral load in EC and cervical cancer is different. The viral load is much lower in EC than in cervical cancer, and commercially available methods and products may not be suitable for HPV detection in EC. In addition, deletion of viral gene fragments and inappropriate gene insertion sites in some cells may fail to express proteins normally, resulting in low protein detection rates in samples. During tumor development, HPV may be cleared by the immune response, and the virus only integrates into host cells after repeated infection, which may also result in undetectable HPV in some samples. Another, it may take a long time for the integrated gene to be expressed as a protein and transform cells into tumors, which is also an important factor affecting detection efficiency. ESCC etiology is also different between high- and low-risk areas. These are all likely causes for differences in research results.

Our study findings indicate that HPV plays an important role in the occurrence and development of EC, but further research is still needed. We used a large number of tissue samples to study the relationship between HPV and EC, as no joint investigation has been conducted in areas with high EC incidence in China. We hope that this study will further confirm the etiological significance of HPV in the development and progression of esophageal squamous cell carcinoma. Clarifying the sources and routes of HPV infections will facilitate the prevention of HPV-positive EC.

## Conclusion

This study was conducted in a region in China with high EC incidence. EC is associated with a high percentage of HPV infections. HPV16 seems to be more easily detected in EC tissues than HPV18. E6 onco-protein is highly expressed in EC tissues and cells and interacts with tumor suppressor protein p53, suggesting that HPV16 may play an important role in the development of EC and that E6 protein may be the key protein of HPV16 affecting EC carcinogenesis. The prevalence of HPV in EC in China suggests that HPV infection may be an important pathogenic factor of EC.

## Supplementary Information

Below is the link to the electronic supplementary material.

Supplementary file1 (DOC 175 KB)

## Data Availability

All data generated or analyzed during this study are included in this published article and its supplementary information files.
