# Supplementary material for: Human papillomavirus is an important risk factor for esophageal carcinoma in a Chinese population
Source: J Cancer Res Clin Oncol. 2022 Nov 17;149(8):5241–53. doi: 10.1007/s00432-022-04322-5 (PMC10349784; doi:10.1007/s00432-022-04322-5)
Supplement: Supplementary file 1 — Supplementary file1 (DOC 175 KB) [file 432_2022_4322_MOESM1_ESM.doc]

# Supplementary Information


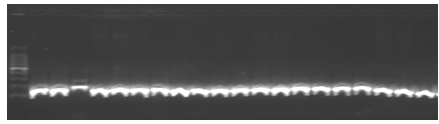


(a)


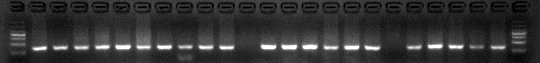


(b)


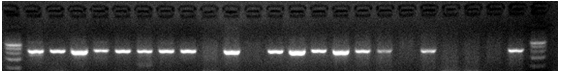


(c)


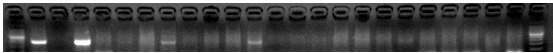


(d)

**Fig. S1** Electrophoresis analysis of PCR-amplified products. (**a**) β-globin PCR. (**b**) GP5+/GP6+ PCR for human papillomavirus (HPV) L1. (**c**) HPV16 E6 PCR. (**d**) HPV18 E6 PCR.
